# Know the Anatomy of Your Intrauterine Device: A Case of Hormonal Sleeve Displacement Resulting in Concerns Over Foreign Body Loss

**DOI:** 10.7759/cureus.85326

**Published:** 2025-06-04

**Authors:** Weerawaroon Mavichak, Mohammed Al Kharfan, Brendan Gallagher

**Affiliations:** 1 Obstetrics and Gynaecology, Royal Surrey County Hospital, Guildford, GBR

**Keywords:** female sexual health, gyne, hormonal contraception, long acting contraception, mirena

## Abstract

Intrauterine devices (IUDs) are one of the most commonly used forms of long-term reversible contraception and are also used for treating gynaecological conditions such as dysmenorrhea, endometrial hyperplasia, and abnormal uterine bleeding. While IUDs have a high safety profile, complications may occur during insertion, use, and removal. IUD fragmentation is a rare but potential complication, in which patients may be subjected to invasive management. We describe a 52-year-old female patient who, after having her levonorgestrel IUD removed, was mistakenly believed to have IUD remnants in her uterus. The removed device was found to be bilaterally armless, subjecting her to hysteroscopy. In her previous removal, the same phenomenon occurred, leading to a pelvic X-ray. It was discovered that the device’s hormonal sheath slid over both arms upon removal, most likely due to a narrowed cervical canal, giving the false appearance of an elongated armless device. To our knowledge, there has been no reported literature on this occurrence apart from being described in the United Kingdom's Faculty of Sexual and Reproductive Healthcare Guideline on Intrauterine Contraception.

## Introduction

Intrauterine devices (IUDs) are one of the most widely used reversible contraception methods due to their safety profile for long-term use, high effectiveness, and affordability [1]. There are currently two main types of IUDs available: copper and levonorgestrel-containing devices. Besides their contraceptive role, hormonal IUDs are also used in treating abnormal uterine bleeding, dysmenorrhea, endometrial hyperplasia, and endometriosis [2]. Rare but serious complications include uterine perforation, migration, pelvic infection, ectopic pregnancies, and fragmentation, which can occur throughout the phases of insertion, during use, and removal [3-5]. IUD fragmentation is defined as the breakage and retention of a component of the IUD inside the uterus, either following removal or spontaneously. Although IUD fragmentation is rare, growing usage of IUDs can lead to an increased rate of such occurrences.

Traditionally, IUDs are T-shaped (framed) with removal threads attached at the bottom end. The licensed duration of usage varies across the types, ranging from 3 to 10 years. At the time of writing, there are five licensed hormonal IUDs in the UK: Mirena, Kyleena, Jaydess, Levosert, and Benilexa. These hormonal IUDs consist of two flexible plastic arms forming the top T-shape, allowing the device to anchor in the uterus, and a white cylinder hormone-containing sheath around the core. Here, we describe a case of an armless hormonal IUD (Mirena) mistaken as IUD fragmentation following removal, subjecting the patient to unnecessary radiography and hysteroscopy.

## Case presentation

**Figure 1:**
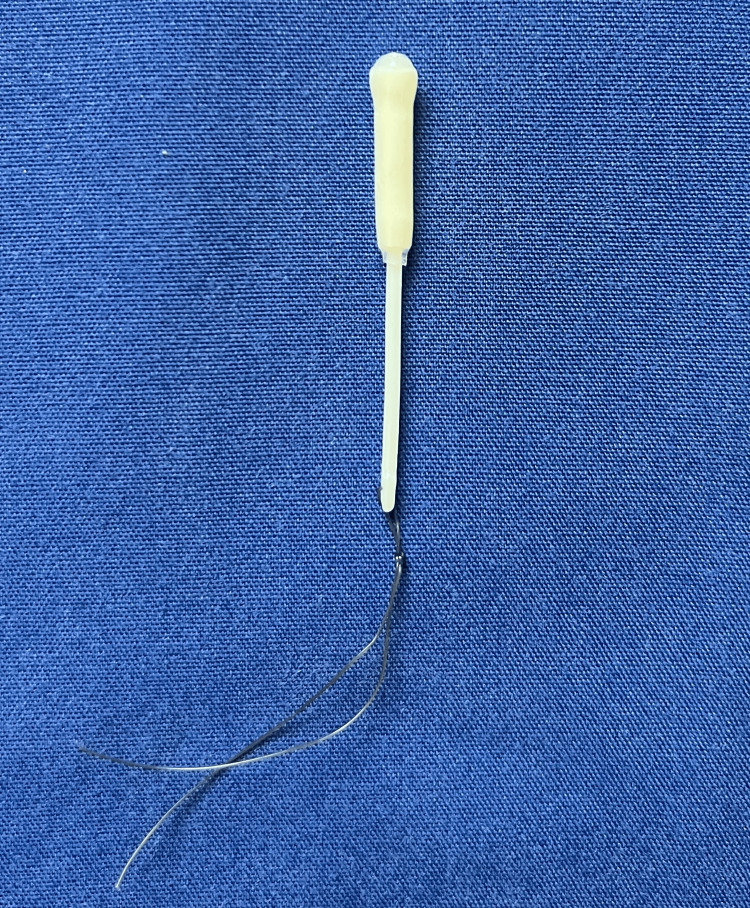
A Mirena device replicated to resemble the shape it was upon removal. The hormonal sheath was slid up, making the arms invisible.

**Figure 2:**
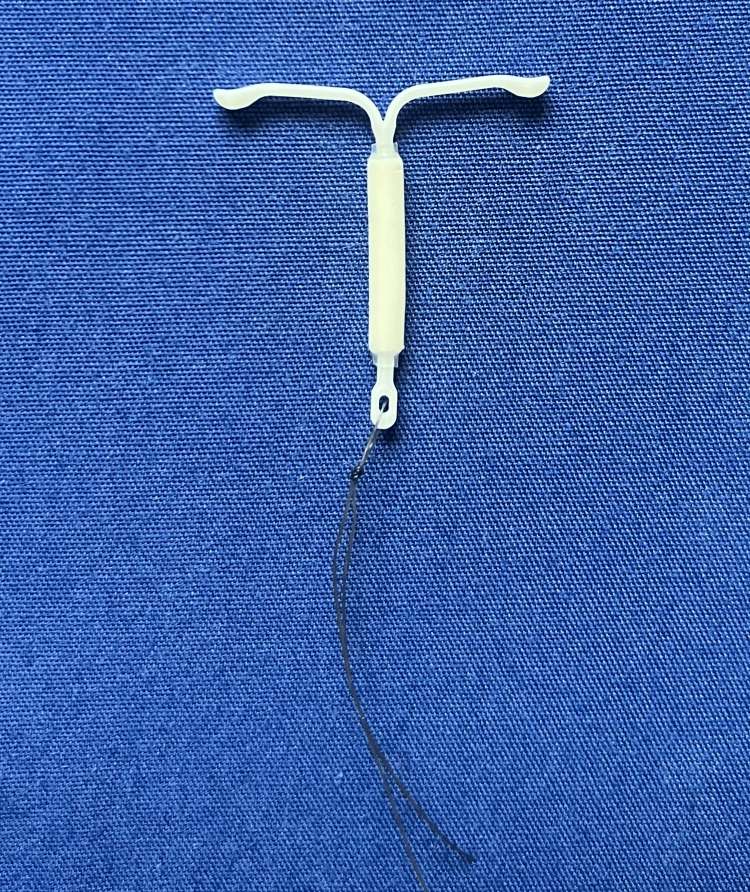
A Mirena device in its intended shape while sitting in the uterus and how it should appear upon removal.

A 52-year-old female patient attended the Day Surgical Unit for routine cervical smear surveillance and IUD removal in June 2024 under general anaesthesia, as per her personal preference. Her obstetric and gynaecological history included three vaginal deliveries, one termination, one miscarriage, and two previous large loop excision of the transformation zone (LLETZ) procedures. Her previous Mirena IUD was inserted in 2019, with no reported complications or complaints of pelvic pain. On the day of the procedure, vaginal examination was normal, and the removal thread was easily identified. The IUD was successfully removed using grasping forceps. Upon removal, we noticed that the IUD was bilaterally armless (Figure 1), compared to what it should have looked like (Figure 2).

We initially deemed that the IUD arms must have fractured inside or become embedded in the uterus. In the patient’s best interest, a hysteroscopy was performed immediately after the IUD removal to search for and retrieve any missing parts. Hysteroscopy revealed a normal and intact endometrium with no signs of device remnants. Upon reviewing the notes, we discovered that the same incident had occurred during her previous IUD removal in 2019, which led to a pelvic X-ray; however, no remnants were seen. Therefore, we examined the removed IUD and attempted to invert the structure by pulling the hormonal sheath downwards, which then revealed the two arms. The removed IUD was compared to a new Mirena IUD, and we concluded that the removed device was intact. We postulate that removal of the IUD through a narrowed cervical canal, likely due to her two previous LLETZ procedures, caused the hormonal sheath to be pulled upwards over the arms, resulting in the misleading appearance of an armless IUD. The patient was informed of the situation while in the recovery bay, and there were no further complications. She was discharged on the same day. The patient consented to the publication of this case.

## Discussion

IUD fragmentation, missing thread, and incomplete removal are recognised complications of IUD removal [5]. IUD fragmentation is a rare mechanical complication that is not well studied and therefore poses a distinct challenge for clinicians [6, 7]. Fragmentation should be considered when the removed IUD is found to have missing components [8]. This finding can provoke anxiety in both patients and clinicians. Cases of IUD arm fragmentation have been reported following both removal and spontaneous expulsion [4, 8-10]. However, it is noteworthy that the majority of fragmentation cases reported involve copper IUDs, not hormonal IUDs. A Food and Drug Administration Adverse Event Reporting System (FAERS) study concluded a distinct disparity in breakage rates between copper and hormonal IUD removals (9.6% and 1.7%, respectively) [11]. Another cross-sectional international study also found that fragmentation occurs more commonly in copper IUDs than in hormonal IUDs [2]. To our knowledge, there are no published cases of an inverted hormonal IUD being mistaken for broken IUD arms, aside from its mention in the UK's Faculty of Sexual and Reproductive Healthcare (FSRH) Intrauterine Contraception guideline [12].

To date, there is no published literature evaluating long-term complications arising from retained IUD fragments, and documentation on this topic remains limited [5, 6, 10, 12, 13]. Some authors recommend timely removal of IUD fragments due to concerns that foreign bodies can provoke inflammatory responses leading to pelvic pain, infection, migration to neighbouring organs, adhesions, bleeding, and infertility [6, 9, 10, 14]. One case study reported no complications after leaving a copper IUD fragment in situ; however, the follow-up was limited to only 12 months [8]. Another case described a patient who presented with secondary amenorrhea 12 months after her Mirena device had been removed and was found to have a retained hormone sheath in her uterus [15].

Management of IUD fragmentation varies across studies [13]. Retrieval methods for IUD fragments upon removal include hysteroscopy, ultrasound-guided forceps, IUD hooks, and suction curettage [2]. The FSRH advises the use of ultrasound or hysteroscopy to locate larger IUD fragments, such as the arms, with radiation imaging if perforation is suspected [12]. Other studies recommend ultrasound and pelvic X-ray as first-line investigations, reserving hysteroscopy for cases where these methods fail due to their invasive nature [13, 16]. Hysteroscopy has been proven to be successful in visualising and removing retained IUD fragments [17]. While some studies have reported indications for laparoscopy, laparotomy, and hysterectomy, a watch-and-wait approach has also been found effective in premenopausal women, based on the rationale that the fragment may migrate with menses [6, 10, 13]. In our case, the patient was already under general anaesthesia, and in her best interest, we proceeded with hysteroscopy given the concern that the IUD was missing its arms. Given the variability in management approaches, patients should receive individualised care tailored to their clinical situation, considering their symptoms and fertility status.

The FSRH Intrauterine Contraception guideline mentions that an IUD may appear elongated and armless upon removal if the hormone sheath slides upward, covering both arms; anecdotally, this can be caused by a narrowed cervical canal [12]. Considering this patient’s history of two previous LLETZ procedures and her age, it is likely that she had a narrowed cervical canal, which caused the hormone sheath to be pulled over the arms during removal. This may explain why the patient experienced two separate instances of an armless IUD after removal. By acknowledging this potential occurrence and being familiar with the FSRH guideline, both in UK practice and potentially in other countries, clinicians can prevent patients from undergoing unnecessary interventions such as X-rays, hysteroscopies, and even laparoscopies.

A case of an elongated and armless IUD should raise suspicion of upward movement of the hormone sheath. This highlights the importance of confirming IUD placement after insertion, ensuring IUD components are intact following removal, and examining the removed IUD thoroughly before concluding fragmentation. A potential suggestion for manufacturers is to consider securing the hormone sheath so that it does not slide upward and cover the arms of the IUD during removal.

## Conclusions

In conclusion, we present a case in which an armless IUD was mistaken for IUD fragmentation during removal. We emphasize the importance of thoroughly examining the IUD components after removal and recognizing the possible occurrence of hormone sheath displacement covering the arms before subjecting patients to invasive investigations. Clinicians who remove hormonal IUDs should be aware of this, particularly in patients with risk factors for a narrowed cervical canal.
